# Serial Determinations of Molecular Aberrations in Patients with Acute Myeloid Leukemia During Treatment with Oral Decitabine/Cedazuridine

**DOI:** 10.3390/cancers18071093

**Published:** 2026-03-27

**Authors:** Klaus Geissler, Gabriele Benetka, Maximilian Prinz-Wohlgenannt, Wolfgang R. Sperr

**Affiliations:** 1 Medical School, Sigmund Freud Private University, Sigmund Freud Platz 3, 1020 Vienna, Austria; 2 Karl Landsteiner Institute for Hematooncological Research, 1020 Vienna, Austria; 3 Institute of Clinical Pathology and Molecular Pathology, Hospital Horn, 3580 Horn, Austria; 4 Department of Hematology and Hemostaseology, Medical University of Vienna, 1090 Vienna, Austria

**Keywords:** AML, decitabine/cedazuridine, NGS, serial determinations, molecular aberrations

## Abstract

Recently, oral decitabine/cedazuridine has been approved for the treatment of AML patients who are not eligible for intensive chemotherapy. Although efficacy data on phenotypic features and the prognostic impact of molecular aberrations at diagnosis were reported in the registration study, serial determinations of the mutational landscape during therapy were not reported. In this study, we present data on a subset of five patients in whom molecular markers were monitored during treatment with oral decitabine/cedazuridine within the registration study. The following observations were made in individual patients. Regarding the changes in the molecular landscape during therapy in four/five patients, there was no major (>50%) reduction in mutated AML clones. There was only one patient with CRi and more than 50% reduction in the VAF of clones with molecular aberrations, including RAS pathway mutations. We observed a marked drop of blast cells (>50%) in two other patients without changes in the molecular profile. The overall survival was significantly longer in patients with CRi and PR, respectively, as compared to patients with no response. Finally, four/five (80%) of patients had druggable molecular aberrations at diagnosis, including mutations in IDH2 (2/5), NPM1 (2/5), and FLT3 (1/5). Our results show that in the majority of patients, changes in the genetic profiles are not seen despite decreases in blast cells in some patients. Disease-modifying activity with decreases in mutated clones is rare. Although the exact mechanism behind our findings remains undetermined, they are in line with the proposed effects of HMA on epigenetics in leukemia cells.

## Simple Summary

Serial determinations of molecular aberrations in patients with acute myeloid leukemia (AML) during treatment with oral decitabine/cedazuridine have not been reported so far. We describe our observations in five AML patients who were treated within the ASCTERAIN trial at our site and had sequential molecular analyses. In four of five patients there were no profound molecular changes whereas major decreases of mutated subclones were seen in one patient suggesting some disease modifying activity of this treatment.

## 1. Introduction

Acute myeloid leukemia (AML) is a disease of the elderly, affecting patients with a median age of 65–70 years [1]. Cytotoxic chemotherapy with or without stem cell transplantation remains the mainstay of treatment in younger patients, but is associated with lower complete remission rates and greater toxicity in older patients, resulting in poor long-term outcomes.

Hypomethylating agents (HMA) such as AZA and DEC +/− Venetoclax are widely used in AML patients who are not fit enough for intensive chemotherapy due to the fact that they have been shown to increase the incidence of remission and overall survival [2,3,4,5,6,7,8,9]. Recently, an oral form of decitabine has been approved in Europe due to the fact that equivalent systemic exposures were demonstrated between the two decitabine formulations in a randomized, crossover, registration, pharmacokinetics study [10].

Knowledge of the molecular and clonal characteristics in AML at baseline and under treatment may be helpful to better understand the disease dynamics and optimize treatment. In a few studies, serial molecular profiling during parenteral treatment with HMA has been done in myelodysplastic syndromes (MDS) and AML, respectively [11,12,13]. However, in patients receiving oral DEC, serial molecular investigations have not been reported so far.

Here, we report on a subset of five patients for whom serial molecular investigations were available during treatment with decitabine/cedazuridine, which was given within the ASTX727-02 trial.

## 2. Patients and Methods

### 2.1. Trial Design

The design of the whole ASCERTAIN trial (ASTX727-02) was reported recently [Geissler].

During the first two 28-day treatment cycles, eligible patients were randomly allocated in a 1:1 ratio to one of two sequences: either oral DEC-C (one fixed-dose combination tablet once daily: decitabine 35 mg/cedazuridine 100 mg) in cycle 1 and DEC-IV (20 mg/m^2^/day, 1 h infusion) in cycle 2 (sequence A), or the reverse order (sequence B). Each treatment was assigned to the first five days of a 28-day cycle. Following cycle 2, patients were treated with oral DEC-C for the first five days of every 28-day cycle until the study was stopped for any number of reasons, including the progression of their disease or intolerable toxicity. Starting with cycle 3, a dose delay (postponing the subsequent cycle) was permitted at the investigator’s discretion in order to recover the blood count from drug-induced myelosuppression.

The study was carried out in accordance with the protocol, the International Council for Harmonization’s Good Clinical Practice guideline, and any applicable local regulations.

Informed consent was given by the patients.

### 2.2. Randomization and Eligibility

An interactive web response system run by Syneos Health (Morrisville, NC, USA) was used to assign patients to treatment sequences based on a computer-generated randomization schedule. It was an open-label study. Eligibility criteria included patients with de novo or secondary AML (as defined by the World Health Organization) [11] who were at least 18 years old, not candidates for standard induction chemotherapy, and who had not received cytotoxic chemotherapy for AML other than hydroxyurea for elevated white blood cell counts. Those who had taken decitabine or azacitidine for more than one cycle were not included. Life expectancy ≥ 3 months and Eastern Cooperative Oncology Group performance status 0 or 1 were additional inclusion requirements.

### 2.3. Molecular Analyses

Serial determinations of molecular aberrations in 5 patients treated in the Austrian site were available for analysis. Genomic DNA was isolated from mononuclear cell (MNC) fractions of bone marrow samples according to standard procedures (QIAamp DSP DNA Blood Mini Kit, Qiagen, Venlo, The Netherlands). The mutational status of AML-related protein-coding genes was determined by targeted amplicon sequencing using the Oncomine Myeloid Assay GX v2 (DNA) (Thermo Fisher Scientific, Waltham, MA, USA). Details regarding the gene panel, library preparation, and data processing are given in the Supporting Information (Methods).

### 2.4. Statistics

According to the 2022 recommendations from an international expert panel on behalf of the ELN response criteria, clinical response was evaluated as complete response (CR), CR with incomplete platelet recovery, CR with incomplete blood count recovery (CRi), or partial response [1]. The log-rank test was used to determine whether individual parameters were associated with overall survival (OS). OS was defined as the time from sampling to death (uncensored) or last follow-up (censored). Dichotomous variables were compared between different groups with the use of the Chi-square test. The Mann–Whitney U test was used to compare two unmatched groups when continuous variables were nonnormally distributed. Results were considered significant at *p* < 0.05. Statistical analyses were performed with SPSS v. 27 (IBM Corp., Armonk, NY, USA); the reported *p*-values were two-sided.

## 3. Results

### 3.1. Patient Demographic and Hematologic Characteristics

**Figure 1:**
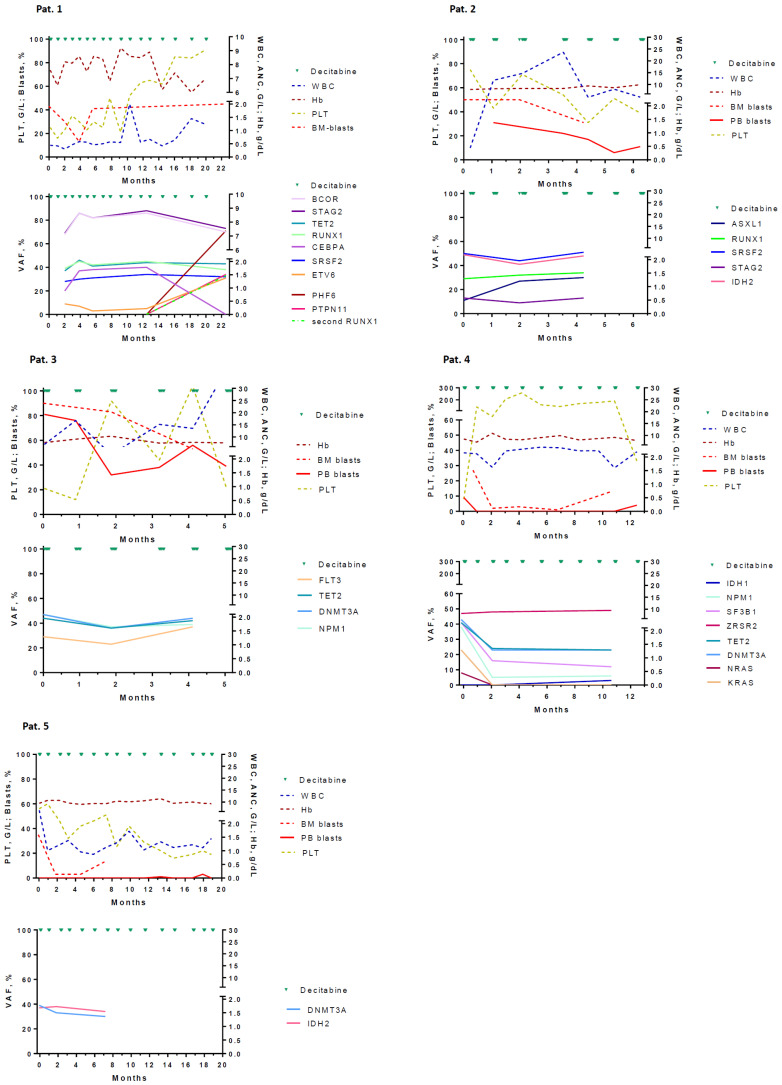
Changes in hematological and molecular parameters in individual patients with acute myeloid leukemia during treatment with oral decitabine/cedazuridine. WBC, white blood cell count; Hb, hemoglobin; PLT, platelets; BM, bone marrow; PB, peripheral blood.

Patient demographics, as well as hematological parameters at diagnosis and at the time of best response, are summarized in Table 1 and Table 2. Moreover, changes in molecular profiles during treatment with oral decitabine/cedazuridine are shown in Figure 1. Serial data were collected from five patients with AML who were not fit enough for intensive chemotherapy and were treated within the ASTX727-02 trial, which was a phase 3, randomized, open-label, crossover study of Cedazuridine and Decitabine fixed-dose combination versus IV Decitabine in Subjects with acute myeloid leukemia (AML).

### 3.2. Case Presentations

Pat 1 was an 84-year-old man who was diagnosed with AML without maturation. He had a history of diverticulosis of the colon complicated by repetitive bleeding. At diagnosis, he showed a normal karyotype, but NGS revealed multiple molecular aberrations, including RUNX1 (Table 1). Two months after initiation of study treatment, bone marrow blast cells decreased by more than 50%, indicating a partial response according to the criteria published by the study group. Despite this phenotypic change, there was no significant change in the molecular landscape for 10 months. At 20 months, bone marrow (BM) examination molecular analysis revealed a clear change in the mutational landscape with the disappearance of the CEBPA subclone but emergence of other subclones with mutations in the genes PHF6, ETV6, and a second RUNX1 clone, indicating clonal evolution. Clinically, the course of disease was characterized by repetitive bleeding episodes from colon diverticula requiring regular blood transfusions. The patient died 24 months after inclusion into the study from his disease.

Pat 2 was a 76-year-old male patient who was diagnosed with AML with MDS-related changes. He had atherosclerosis, namely coronary artery disease with STENT implantation 2 years ago and cerebrovascular disease with an infarction of the thalamus 4 years ago. BM examinations at months 2 and 6 of therapy showed blast cell persistence without signs of response. The patient died 8 months after inclusion into the study from his disease.

Pat 3 was an 81-year-old woman who was diagnosed with AML with mutated NPM1. She had chronic renal failure with a glomerular filtration rate of 38 mL/min. Molecular analysis revealed, in addition to mutated epigenetic genes like DNMT3A and TET2, molecular aberrations in the NPM1 gene and the FLT3 gene. In her BM, she had 90% blasts at diagnosis. BM examinations showed treatment failure at months 2 and 4. The patient died 6 months after initiation of therapy. The molecular landscape did not show major changes during treatment.

Patient 4 was an 84-year-old woman who was diagnosed with AML with mutated NPM1. Because of leukocytosis (WBC 83.5 × 10^9^/L), she received hydroxyurea before starting treatment with decitabine. Except for arterial hypertension, she had no comorbidities. After 2 months of therapy, the BM examination showed a decrease in the BM blast cells to 2%. In the peripheral blood, a rise in the platelet count to 78 G/L was observed, but no increase in ANC. Thus, the response was classified as CRi according to ELN criteria. Interestingly, VAF of mutated SF3B1, IDH1, TET2, and DNMT3A dropped by >50% as compared to baseline values, and KRAS and NRAS subclones completely disappeared. Only the VAF of the ZRSR2 mutation persisted unchanged during treatment, which may be considered as an indication of clonal hematopoiesis before the development of AML in this patient. BM aspiration at 12 months showed a re-increase of blast cells to 14%. There was no change in the molecular landscape at that time as assessed by NGS. Following the recurrence of the disease, the patient died after 15 months of therapy.

Patient 5 was an 88-year-old female patient who was diagnosed with AML with myelodysplasia-related changes. Comorbidities included polyarthritis, diverticulosis of the colon, and osteoporosis. The patient had a normal karyotype. Molecular analyses showed mutations in DNMT3A and IDH2. Two months following the initiation of treatment, the percentage of blast cells in the BM dropped to 3%. However, the cytopenia in the PB persisted, indicating CRi. Molecular analysis of BM cells at 2 and 6 months showed no significant change in the mutation pattern. In the following BM examinations at 4 and 12 months, the blast cell percentage increased to 10% and 15%, respectively. Thirty-two months after the beginning of treatment, the patient died from her disease.

### 3.3. Changes in Blast Cell Percentages and Molecular Landscape

**Figure 2:**
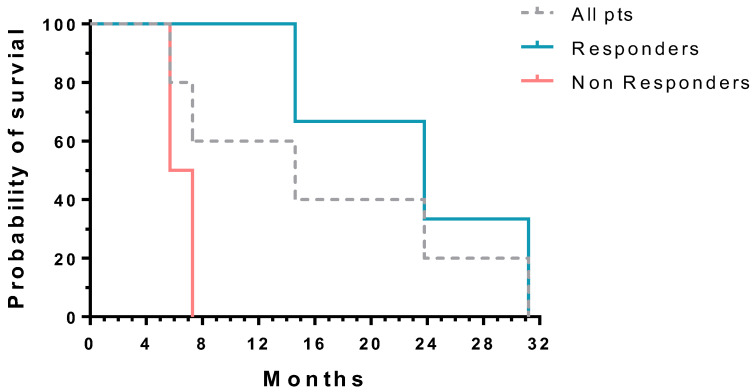
Overall survival of AML patients with response (PR, CRi) and without response treated with oral decitabine/cedazuridine. The difference in survival between responders and non-responders was statistically significant as assessed by the log-rank test (*p* = 0.039).

Changes in blast cell percentages at diagnosis and during treatment are shown in Table 2. Moreover, changes in the molecular landscape in individual patients during treatment are shown in Figure 1. Using the ELN AML response criteria defined in 2017 [1], two patients had CRi, one patient had PR, and two patients had no response. The overall survival was significantly longer in patients with CRi and PR, respectively, as compared to patients with no response (Figure 2). Regarding the changes in the molecular landscape during therapy, there was only one patient with more that 50% reduction in the VAF of clones with molecular aberrations, including RAS pathway mutations (Pat 5). In this patient, the clear molecular response in association with CRi suggested a disease-modifying effect of therapy. However, we observed a marked drop (>50%) of blast cells in two patients without changes in the molecular profile (Pat 1 and 4). In the total cohort of the five patients, there was no significant difference in survival regarding molecular changes (Suppl Figure S1). In one patient, we could demonstrate clonal evolution before the progression of the disease. Finally, it is important to mention that four/five (80%) of patients had druggable molecular aberrations at diagnosis, including mutations in IDH2 (2/5), NPM1 (2/5), and FLT3 (1/5).

## 4. Discussion

AML is a genetically heterogeneous and complex disease in which the molecular pattern may change during the course of the disease due to disease evolution and/or the impact of treatment. Investigating the clonal characteristics in AML at baseline and under treatment may be helpful to better understand the disease dynamics and optimize treatment.

Our major finding was that in the majority of patients, changes in the genetic profiles are not seen despite decreases in blast cells in some patients. This is in agreement with findings in patients with chronic myelomonocytic leukemia (CMML) in whom mutation allele burden remained unchanged even in patients responding to hypomethylating agents [11]. AML is an extremely heterogeneous disease, and there are several possibilities to explain decreases in blast cells without major changes in molecular aberrations. Currently, one of the widely used proposed explanatory models includes demethylation of aberrantly silenced genes leading to the reactivation of tumor-suppressor genes or genes involved in differentiation [12]. However, there are also alternative explanations that have to be considered. Several mutations tracked in our patients, including DNMT3A, TET2, ASXL1, and ZRSR2, are well-established markers of clonal hematopoiesis of indeterminate potential (CHIP) and can persist in non-leukemic cells entirely independent of treatment response [13,14]. Thus, stable VAF during blast reduction may simply reflect the persistence of pre-existing CHIP clones rather than any epigenetic drug effect. This is a possible explanation for patients carrying the mutations mentioned above, but it is unlikely to be the only explanation for patients with mutated subclones. Transient differentiation effects, sampling variability, or shifts in subclonal dynamics below the detection threshold could also be alternative explanations.

The impact of HMA on mutational profiles has also been reported by other groups. Symeonidou et al. reported that heterogeneous genetic and non-genetic mechanisms contribute to response and resistance to azacitidine monotherapy [15]. In responders, three types of response were observed, including an almost complete elimination of mutations in 33%, no change in 17%, and changes with no discernible pattern in 50%. In a study reported by Uy et al., responding patients, including those in CR, could have persistent measurable tumor burden for at least 1 year without disease progression [16]. Using ultrasensitive sequencing, extremely rare mutations could be detected months to years before their expansion at disease relapse. Although patients could live with persistent clonal hematopoiesis in a CR or stable disease, ultimately, expansion of a rare subclone occurred at relapse or progression. In a study by Welch in which AML and MDS patients received decitabine IV at a dose of 20 mg per square meter of body-surface area per day for 10 consecutive days in monthly cycles, clearance of leukemia-specific mutations correlated closely with morphologic and cytogenetic responses [17]. In samples obtained from 20 patients with blast clearance in the bone marrow with or without peripheral recovery after day 28 of cycle 2, the authors were able to detect leukemia-specific mutations despite morphologic remission. This indicated that decitabine leads only to incomplete clearance of the disease. There was no difference between patients with or without peripheral blood count recovery with regard to clearance of leukemia-specific mutations or the duration of remission.

Marked changes in the mutation profile by HMA are rarely reported in the literature. However, in one of our patients, we observed a marked decrease or even clearance of multiple mutations, including NPM1, TET2, SF3B1, KRAS, and NRAS, whereas the VAF of the molecular aberration of the ZRSR2 gene persisted. The impact of treatment on the genetic complexity indicates some disease-modifying potential of this therapy. Because of leukocytosis (WBC 83.5 × 10^9^/L), she received hydroxyurea before starting treatment with decitabine as required in the protocol. Thus, we cannot completely rule out that prior HU treatment may have had some impact on subsequent decitabine effects, but this has not been systematically studied so far. Disease-modifying activity has been shown to be associated with survival benefit in other clonal myeloid diseases, including chronic myeloid leukemia, primary myelofibrosis, and polycythemia vera [18,19,20]. Thus, the disease-modifying activity of oral decitabine/cedazuridine seems to be a rare event but may be observed in a few patients. Therefore, predictive markers that could identify these patients would be of major significance.

The effect of DEC on subclones with RAS pathway mutation is another interesting finding in this patient. Since RAS pathway mutations are commonly found in other malignancies, this observation may provide a rationale to integrate HMA in treatment concepts for other tumor entities [21]. In fact, HMA has been shown to be effective in solid tumors in the preclinical setting [22,23].

Clonal evolution could be demonstrated in one of our patients. In this phenomenon, new subclones emerge, whereas other clones may be suppressed or even may disappear completely. Changes in the molecular landscape may be explained by differences in the molecular fitness of different subclones. Since we did not have molecular analyses in all our patients in the terminal phase of their disease, we cannot estimate the proportion of clonal evolution in our patients. Lack of clonal evolution between remission and relapse has been reported in some AML patients receiving azacitidine, again suggesting that non-genetic mechanisms might be involved in these patients [15].

The OS of responders was longer than that of non-responders. This is in agreement with other studies using HMA in AML and confirms the activity of these agents in the treatment of this disease [5,7]. On the other hand, there was no significant difference in survival regarding molecular changes. One possible explanation for this discrepancy between morphological and molecular response could be that hematopoiesis may be reconstituted by mutated stem cells in some patients. These cells carry mutations but produce differentiated progeny. In this case, molecular signals persist, but morphology appears improved. Such clonal remissions have been reported previously in AML patients treated with chemotherapy [24].

There are obviously a number of inherent limitations of this study. Therefore, our observations should be mainly regarded as exploratory or hypothesis-generating rather than as evidence supporting broader mechanistic or clinical conclusions. These limitations include the small number of patients and the fact that AML is a biologically heterogeneous disease where clonal evolution and therapeutic responses vary substantially among patients. Individual variability may reflect patient-specific trajectories rather than reproducible biological patterns. Moreover, the cohort itself is heterogeneous in terms of age, comorbidities, cytogenetic background, and baseline molecular profiles, introducing additional sources of variability, reducing the statistical power, restricting its external validity, and generalizability of the findings.

Serial determinations of molecular aberrations in AML patients treated with oral decitabine/cedazuridine have not been published so far. Oral therapy for older, often infirm patients with AML ineligible for intensive chemotherapy may provide significant benefit over parenteral treatment. The safety profile with oral decitabine/cedazuridine was consistent with that previously observed for intravenous decitabine (Suppl Table S1). Due to the fact that with the combination of decitabine/cedazuridine and venetoclax, a fully oral AML therapy will be available for a large number of patients in the future, one can expect that information about the impact on the molecular profiles of patients treated with decitabine/cedazuridine will become increasingly important.

Our data also show a relatively high prevalence of druggable mutations, including molecular aberrations. In our series, we have seen FLT3, IDH2, and NPM1 mutations that might be targetable with drugs such as midostaurin [25], enasidenib [26], and ziftomenib [27], respectively, but which were not approved for combinations with HMA at the time of study. Considering the profound improvement of OS in IDH1-mutated AML patients by the addition of ivosidenib to HMA, combination strategies in these patients certainly deserve further attention [28].

## 5. Conclusions

In conclusion we observed no major changes in in the genetic profiles in the majority of patients treated with oral decitabine/cedazuridine in line with the proposed effects of HMA on epigenetics in leukemia cells. A larger cohort of AML patients needs to be studied to confirm our preliminary results.

## Figures and Tables

**Table 1:** Patient characteristics.

ID	Sex	Age	WHO Cat *	Comorbidities	Cytogenetics	NGS
1	Male	84	AML without maturation	Diverticulosis N prostatae	46XY	RUNX1 39% SRSF2 28% STAG2 69% BCOR 68% TET2 37% ETV6 9% CEBPA 20%
2	male	76	AML with myelodysplasia-related changes	Cardio and cerebrovascular disease	Complex karyotype FISH TP53 loss	IDH2 49% SRSF2 50% ASXL1 11% RUNX1 29% STAG2 13%
3	female	81	AML with mutated NPM1	Chronic renal failure	NA	NPM1 47%, DNMT3A 47% FLT3 29% TET2 44%
4	female	84	AML with mutated NPM1	Arterial hypertension	No growth FISH no aberrations	NPM1 38%, KRAS 23% NRAS 8% TET2 41% SF3B1 41% DNMT3A 43% ZRSR2 47%
5	female	88	AML with myelodysplasia-related changes	Polyarthritis, osteoporosis, and diverticulosis	46XX	DNMT3A 39% IDH2 37%

* According to the WHO classification criteria 2016 [1], AML—acute myeloid leukemia.

**Table 2:** Hematological parameters at diagnosis (pre) and time of best response (post).

ID	BM Blasts pre (%)	BM Blasts Post (%)	ANC pre (G/L)	ANC Post (G/L)	Hb pre (g/dL)	Hb Post (g/dL)	PLT pre (G/L)	PLT Post (G/L)	Best Response	OS Months
1	42	11	0.1	0.24	7.6	8.6	25	30	PR	24
2	50	50	1.4	0.372	7.9	8.5	75	88	NR	8
3	90	80	0.12	0.05	7.7	7.8	21	104	NR	6
4	27	2	0.51	0.275	6.9	10.9	8	78	CRi	15
5	35	3	7.0	0.019	9.5	9.1	56	49	CRi	32

BM, bone marrow; ANC, absolute neutrophil count; Hb, hemoglobin; PLT, platelets; NR, no response; PR, partial response; CRi, complete response with incomplete hematological recovery (according to 2017 ELN recommendations [1]).

## Data Availability

Data available on request due to privacy/ethical restrictions.

## Supplementary Materials

The following supporting information can be downloaded at: https://www.mdpi.com/article/10.3390/cancers18071093/s1, Methods; Table S1: treatment-emergent adverse events regardless of relation to treatment; Figure S1: overall survival of patients with and without molecular changes.
