# Supplementary material for: Serial Determinations of Molecular Aberrations in Patients with Acute Myeloid Leukemia During Treatment with Oral Decitabine/Cedazuridine
Source: Cancers (Basel). 2026 Mar 27;18(7):1093. doi: 10.3390/cancers18071093 (PMC13072061; doi:10.3390/cancers18071093)

Supporting Information

Methods

The Oncomine Myeloid Assay was designed to profile 45 key target genes that frequently harbour mutations in myeloid malignancies. Library preparation and sequencing were conducted in a fully automated manner using the Ion Torrent Genexus System, Thermofisher Scientific.

Full gene sequencing was performed for: ASXL1, BCOR, CALR, CEBPA, ETV6, EZH2, IKZF1, NF1, PHF6, PRPF8, RB1, RUNX1, SH2B3, STAG2, TET2, TP53, ZRSR.

Hotspot sequencing was performed for: ANKRD26, ABL1, BRAF, CBL, CSF3R, DDX41, DNMT3A, FLT3, GATA2, HRAS, IDH1, IDH2, JAK2, KIT, KRAS, MPL, MYD88, NPM1, NRAS, PPM1D, PTPN11, SMC1A, SMC3, SETBP1, SF3B1, SRSF2, U2AF1, WT1

Variant calling:

Mutational analysis was perfomed using the Ion Torrent Genexus Software v.6.8.4.0. Nomenclature of variants followed HGVS guidelines. Variants were classified using the ClinVar database as well as the reference population database gnomAD. Variants with a gnomAD allele frequency >0.01 were considered as benign variants and were thus not reported. If no entry was available in the ClinVar database and allele frequency in gnomAD was <0.01 variants were categorized as variants of uncertain significance (VUS).

Relevant reference transcripts:

CBL: NM_005188.4
ETV6: NM_001987.5
JAK2: NM_004972.4
KIT: NM_000222.3
PPM1D: NM_003620.4
SH2B3: NM_005475.3

Suppl. Table S1

≥ Treatment-emergent adverse events regardless of relation to treatment

Toxicity	Pat 1	Pat 2	Pat 3	Pat 4	Pat 5
Thrombocytopenia	+	+	+	+	+
Anemia	+	+	+	+	+
Neutropenia	+	+	+	+	+
Febrile Neutropenia			+		
Infection		+		+	+
Bleeding	+	+	+		+
Asthenia	+		+	+	+
Diarrhea				+	+
Nausea			+		
Dyspnea			+		+
Bonepain/Arthritis				+	+
Edema					+

Suppl. Figure S1

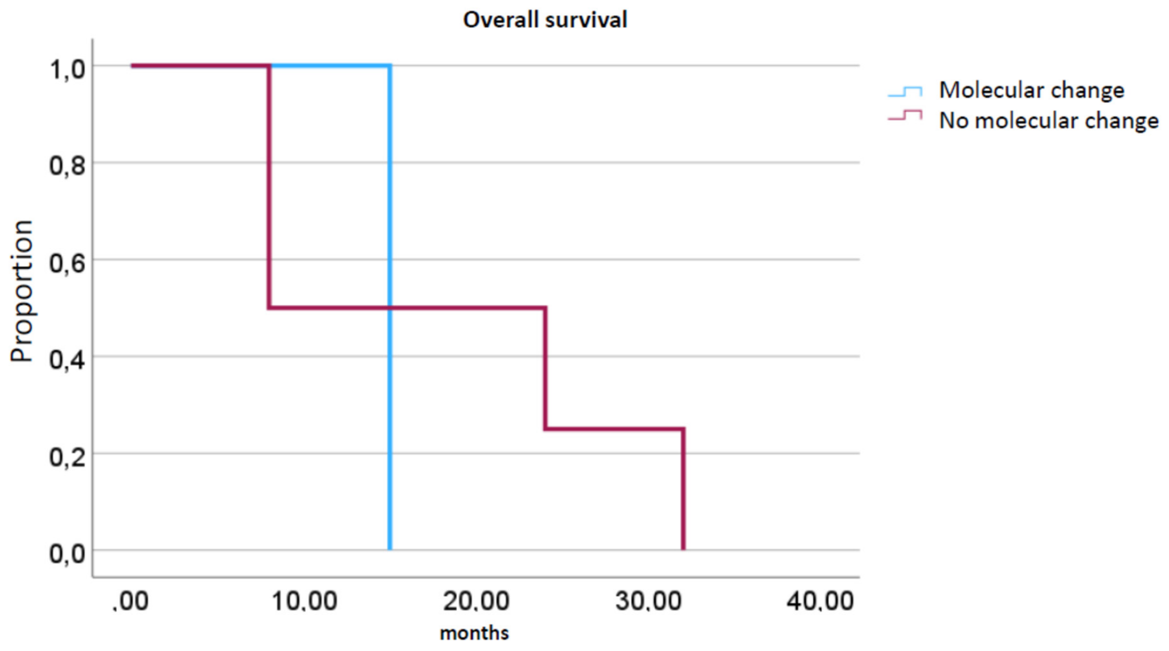

Supplement: Supplementary file 1 [file cancers-18-01093-s001.zip › cancers-4205252-supplementary.pdf]
